# Two Cases of Luxation of the Elbow Backward

**Published:** 1871-04

**Authors:** Lewis A. Sayre

**Affiliations:** Professor of Orthopedic and Clinical Surgery, Bellevue Hospital Medical College


### TWO CASES OF LUXATION OF THE ELBOW BACKWARD—ONE OF 14 AND THE OTHER OF 16 WEEKS' STANDING—BOTH SUCCESSFULLY REDUCED BY THE AID OF SUBCUTANEOUS SECTION OF THE TRICEPS TENDON, AND RECOVERY WITH NEARLY PERFECT MOTION.*

BY LEWIS A. SAYRE, M. D.,

Professor of Orthopedic and Clinical Surgery, Bellevue Hospital Medical College.

:o:

CASE I.—Richardo Gibbs, æt. 5, years, a native of Cuba, came to me on the 9th of August, 1867, with a luxation of the foream backward, the result of a fall received fourteen weeks previously. The limb was very nearly straight, and fixed at the joint, no motion being manifest. Several attempts had been made in Cuba to reduce it, but they were all unsuccessful. Col. Lewis Lay, of Havana, then sent him to me, and, after a careful examination of the case, I decided that it was worth a trial, although the fibrous adhesions were so firm as to give it very much the appearance of bony anchylosis.

Dr. Frank Hamilton, and Prof. Jas. M. Bush, of Kentucky, were called in consultation, who both agreed with me as to the propriety of attempting the reduction.

The boy was perfectly anæsthetized with chloroform by Dr. Paine, and I made vigorous efforts at reduction by flexion and extension for nearly half an hour, without any manifest change in the position of the bones, although I succeeded in breaking considerably the fibrous adhesions.

Dr. Hamilton then tried the reducton, but without any better result than my first effort. I made another attempt, and finding that I could not succeed unless I fractured off the olecranon, I requested Dr. Hamilton to divid the triceps tendon subcutaneously, which he did, while I made it very tense by my strong flexion, and in a few minutes I succeeded in making the reduction perfect and complete.

The elbow was secured at a right angle with Ahl's adaptable porous-felt splint, and kept wet with an evaporating lotion.

The advantage of these splints over all others that I have

* Read before the Medical Society of the State of New York, at its annual meeting, January 9th, 1871, and ordered to be published in its Transactions.

seen in cases where we wish accuracy of adjustment, with permanency of retention, and at the same time wish to keep the parts wet with lotions, either hot or cold, without any danger of their change of form or position, can only be appreciated by those who have had practical experience in their application. In this case it was retained on the arms for several days, without the slightest change in position, although saturated with the lotion all the time, and gave the greatest possible comfort to the patient.

Two days after the operation the arm was much swelled and greatly discolored from the extravasated blood, but it gradually subsided, and on the tenth day the swelling had almost entirely disappeared, but the discoloration lasted for some weeks.

After the tenth day the splint was removed daily, and passive motions given to the joint, with friction to the arm and forearm. And on the twentieth day from the operation the flexion and extension of the elbow could be made complete and without any pain whatever. There was very little voluntary power over the brachialis anticus muscle, and electricity was applied to it and the biceps flexor.

This was continued every other day for two months, with friction and passive motion to the joint, by which time he could flex his arm to a right angle, could tie and untie his cravat, and extend his arm nearly straight. He then left for his home, and I have not seen him since, but learned from his father, a few months since, that the motions of his elbow were as good as that of the other.

Case II.—On the 7th of December, 1870, Dr. E. S. Dunster sent to my clinic, at the Charity Hospital, a lad æt. 13 years, who had a luxation of his elbow backward, of thirteen weeks' standing, and had been treated by a druggist in the country, by a straight splint, and of course his arm was quite firmly anchylosed in a perfectly straight position.

Dr. Dunster had tried a few days before to reduce the luxation, but without success, and sent him to me for another effort, if I deemed it advisable. The joint was so inflammed at the time from the result of this effort, that I was afraid to attempt it, and advised ice bags to the part until the inflamation should subside. He was presented again on the following week, and while examining his arm, and before any attempt was made to reduce it, he swooned and was revived with great difficulty. The boy was anæmic from starvation, and the bruit in his neck was like that of a chlorotic girl.

As any anæsthetic in his condition was dangerous, and as it was impossible to perform the operation without it, he was advised to wait until his general health was improved, and for that purpose he was put upon a nutritious diet, with a full allowance of iron and quinine.

Some doubts having been expressed as to the propriety of attempting to reduce the bone, I called a consultation on the 22d of December, 1870. Dr. Hewett, formerly surgeon U. S. A., advised exsection—some of the other gentlemen also advised this course. Dr. Mason detected the fracture of the coronoid process, which had become attached to the anterior portion of the condyle of the humerus. The arm was perfectly straight, and admitted of only the slightest possible motion.

After carefully weighing all the points in the case, I decided not to resect it, as it might be followed by tedious suppuration, and his condtion was too feeble to take that risk.—Resection of a joint that has been a long time inflamed, and where the periosteum has become thickened, and will readily peel from the bone, so that you can make the exsection *sub-periosteally* is a very easy matter; but when the bones are in their normal condition, it is impossible to detach the periosteum, except in shreds; and I therefore decided to attempt the reduction, and if it was found impossible to do it, that I would then fracture the olecranon, and take my chances for motion; but at all events have recovery with the arm anchylosed at a right angle, which would make it much more useful than in its present position.

He was fully anæesthetized by ether and with some considerable force I succeeded in breaking up the adhesions and flexing the arm to an angle of about 135°, when I was checked by the tension of the triceps, and fearing that the olecranon would fracture if any more force was used, I requested Dr. Mason to divide the triceps, which he did subcutaneously, and almost instantly the forearm was brought to a right angle with the arm, when it was checked from any other flexion by the coronoid process, which was attached to the anterior portion of the condyle of the humerus. By using the forearm as a lever, and with some considerable force, I fractured off this attachment, and succeeded in flexing the arm to an angle of 45°. The arm was secured in this position and treated the same as in the first case. Very little inflammation followed, and at the end of a fortnight passive movements were commenced, and I was informed yesterday by Dr. Dunster that he has every prospect of a recovery with very good, if not perfect, motion. The dislocation had existed sixteen weeks, lacking two days.

Gross, vol. ii. p. 141, says: "The reduction of this dislocation is extremely easy, if attended to immediately after its occurrence, but very difficult if it be neglected even for a short time.

"Upon this subject there is no difference of sentiment among practitioners, writers and teachers.

"My experience in regard to it is ample and in perfect accordance with that of the profession generally. I have no recollection of ever being foiled in my efforts in a solitary instance of recent dislocation of the elbow joint. While I can recall to mind a large number of cases where everything that could be done proved unavailing after the third week and sometimes even by the end of the second. I am not prepared to assign any reason for this; to say why a displacement that is always so easily rectified, if properly managed in its early stages, should so soon become utterly resisting, and defying all the best directed efforts of the surgeon."

These two cases show that we may sometimes be successful, even after a longer period than three weeks, and therefore justify the effort.

That there was a fracture of the coronoid process in this case, there can be no possibility of a doubt. Its crescentic edge could be distinctly felt on the anterior surface of the condyles of the humerus, and to which it had become quite firmly attached. And when the forearm was strongly flexed, it was fractured off with a very distinct snap, which was clearly audible to the entire class.

The olecranom was not fractured, nor any other bone, and, therefore, this distinct noise of a fracture must have been made by the fracture of the coronoid from its new attachment.

Hamilton, in his work on Fractures, edition 1863, p. 267, *et seq.*, says:

"Dissections have established the possibility of this fracture as a simple accident in the living subject; but I have not myself seen any example of which I can speak positively."

He relates two cases in which the existence of such a fracture was at first suspected, but in regard to which he afterward had very little doubt that his diagnosis was incorrect. After analyzing the reported cases, he concludes:

"The fact, therefore, that so few cases have been reported, and that most of these are far from being clearly made out, remains presumptive evidence that the actual cases are extremely rare. But if to this we add such negative evidence as is furnished by actual dissections and examinations of the pathological cabinets of the world, we think the testimony almost conclusive."

"Only four specimens have been mentioned by any of the surgical writers known to me," and these four he shows to be not altogether satisfactory, and concludes: "We are therefore left as before with no evidence that the coronoid process was ever broken by the action of a muscle, and with only one example in which it is probable a fracture occurred as a consequence of a dislocation of the radius and ulna backward."

The last one of my two cases I feel quite confident can be added to this list, and Dr. Mosely, Demonstrator of Anatomy in Bellevue Hospital Medical College, showed me a specimen, a few days since, which he had obtained in the dissecting room, in which there was distinct fracture of the coronoid process, and union by ligamentous junction.

Dr. Darling, Professor of Anatomy in the Medical University of New York, has another specimen, which was presented to him by Dr. Bradley, of this city, which is almost the exact counterpart of my second case—luxation of both bones backward, and fracture of the coronoid process—which had become anchylosed to the anterior portion of the condyle of the humerus.

The rarity of this accident, according to Hamilton, and the difficulty of its treatment, according to Gross, and which in this instance was so pefectly successful, has appeared to me a suficient reason for placing the facts before the society for permanent record.—*Phil. Medical & Surgical Reporter.*

285 5th *Av.*, *January* 20, 1871.

